# The synergistic antitumour effect of multi-components from *Pulsatilla chinensis* saponins in NCI-H460 lung cancer cell line through induction of apoptosis

**DOI:** 10.1080/13880209.2020.1761404

**Published:** 2020-06-01

**Authors:** Ziyi Guan, Lanying Chen, Yihan Zhou, Yingying Luo, Yaru Cui, Ronghua Liu, Binyao Shou

**Affiliations:** a National Pharmaceutical Engineering Center for Solid Preparation of Chinese Herbal Medicine, Jiangxi University of Traditional Chinese Medicine, Nanchang, Jiangxi, China; b School of Pharmacy, Jiangxi University of Traditional Chinese Medicine, Nanchang, Jiangxi, China

**Keywords:** Antiproliferative, annexin V-FITC, bioinformatic analysis, natural products, proteome, synergism

## Abstract

**Context:**

*Pulsatilla chinensis* (Bunge) Regel (Ranunculaceae) possess antitumour effects; however, its antitumour potential has not been extensively investigated.

**Objective:**

To investigate the synergetic effect of multi-components from *P. chinensis* induced cell apoptosis and explore the mechanism.

**Materials and methods:**

The cytotoxicity was measured against NCI-H460, SMMC-7721, HCT-116 and U251 cell lines treated with eight monomers from *P. chinensis.* The synergetic effect of a combination of Pulsatilla saponin D (PSD), Raddeanoside R13 (R13), and Pulsatilla saponin A (PSA) was assessed using CalcuSyn3.0. Annexin V-FITC/PI and DAPI staining analyzed apoptosis of NCI-H460 cells treated with PSD, R13 and PSA alone or in combination. Proteins differential expression was analyzed using proteomic, DAVID Bioinformatics Resources, R software environment and KEGG database, and verified by western blotting.

**Results:**

PSD, R13, and PSA displayed greater antitumor activity with IC_50_ values of 5.6, 5.1 and 10.5 µM against NCI-H460 cells compared with other monomers. The combination of PSD, R13, and PSA had a synergistic effect at CI = 0.27 and induced 17.53% cells apoptotic detected by flow cytometric. Bioinformatic analysis showed an overview of the differentially expressed proteins and some signalling pathways. Moreover, some candidate proteins (LDHA, PI3K, NOL3 and cleaved-caspase-3) were validated by western blotting.

**Discussion and Conclusion:**

These results show PSD, R13, and PSA are good candidates as natural products for use in the treatment of lung cancer. Potential signalling pathways and protein targets need to be further validated. The application of the drug combination approach also provides a therapeutic strategy for cancer.

## Introduction

Lung cancer is the leading cause of cancer-related deaths among humans worldwide. In the histopathological classification, approximately 75–80% of lung cancers are non-small cell lung cancer (NSCLC) (Hoffman et al. 2000; Siegel et al. 2012). Currently, the treatments for NSCLC include surgery, chemotherapy, and radiotherapy. Chemotherapy has a considerable therapeutic effect and improves the quality of life of patients. However, chemotherapeutic drugs also seriously damage normal cells and cause drug resistance of tumour cells, which has always been an obstacle to the clinical application of drugs (Xu et al. 2010; Yu et al. 2013). Thus, the development of new therapeutic drugs for lung cancer is important.

The root of *Pulsatilla chinensis* (Bunge) Regel (Ranunculaceae) has been used to treat intestinal amoebiasis, vaginal trichomoniasis, malaria, and infections (Cheng et al. 2008; Xu et al. 2012). During recent years, saponins, the main active component from *P. chinensis*, have been extracted, separated, and identified (Ye et al. 1996). Numerous studies have reported that some monomers of *P. chinensis* extract can inhibit the growth of various tumour cells (Zheng et al. 2010; Son et al. 2013; Liu et al. 2014; Liang et al. 2016). However, the anti-proliferative function and the mechanism of action of most *P. chinensis* saponin monomers against NSCLC are unclear. It is known that the therapeutic action of traditional Chinese medicines does not involve just one component. Treatment enhancement and mutual restriction among various drugs or components are the most important features of traditional Chinese medicine. Therefore, a combination of monomers can be considered to overcome drug resistance and reduce toxicity.

**Figure 1.**
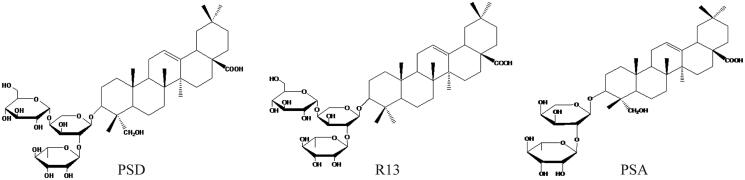
Chemical structures of Pulsatilla saponin D, Raddeanoside R13 (R13) and Pulsatilla saponin A.

In this study, we hypothesized that the three monomers (Pulsatilla saponin A, Raddeanoside R13, and Pulsatilla saponin D) (Figure 1) of *P. chinensis* might exert synergistic effect and thus be more effective than either agent administered alone. We compared the anti-proliferative effect of *P. chinensis* monomers individually and in combination and investigated the potential molecular mechanisms of the anti-proliferative effect of combined monomers against NSCLC using a proteomic analysis.

## Materials and methods

### Reagent and materials

Anemoside A3 (A3), Pulsatilla saponin D (PSD), anemoside B4 (B4), hederacoside C (HC), hederacoside D (HD), and hederagenin (HG) were purchased from Nanjing Spring & Autumn Biological Engineering Co., Ltd. (Nanjing, China). Raddeanoside R13 (R13) and Pulsatilla saponin A (PSA) were provided by the National Pharmaceutical Engineering Centre for Solid Preparation in Chinese Herbal Medicine (Nanchang, China). The purity of all the compounds was greater than 98%. All compounds were dissolved in dimethyl sulfoxide (DMSO) (Sigma, St Louis, MO) and stored at -80 °C. The final concentration of DMSO used in culture media was 0.1% or less. Cisplatin was purchased from Hansoh Pharmaceutical Co., Ltd. (Lianyungang, China). All compounds were diluted in the corresponding culture medium to the desired concentrations.

Roswell Park Memorial Institute (RPMI) 1640 and Dulbecco’s Modified Eagle medium (DMEM) were purchased from Solarbio Science & Technology Co., Ltd. (Beijing, China). Foetal bovine serum (FBS) was purchased from Gibco (Grand Island, NY). 3-(4,5-Dimethyl-2-thiazyl)-2,5-diphenyl-2H-tetrazolium bromide (MTT), trypsin, 4′-6-diamidino-2-phenylindole (DAPI) Staining Kit, paraformaldehyde, dithiothreitol (DTT), indoleacetic acid (IAA), NH_4_HCO_3_, and trifluoroacetic acid (TFA) were obtained from Sigma (St Louis, MO). The Annexin V-FITC/PI apoptosis detection kit was purchased from BD Pharmingen (San Diego, CA). Antibodies against LDHA, PI3K, and NOL3 were purchased from Abcam (Cambridge, UK). Antibodies against β-actin and caspase-3 were obtained from Cell Signalling Technology (Beverly, MA). Bicinchoninic acid (BCA) protein assay kit, anti-rabbit and anti-mouse secondary antibodies, radioimmunoprecipitation assay buffer (RIPA), and phenylmethylsulfonyl fluoride (PMSF) were purchased from Beyotime Institute of Biotechnology (Shanghai, China). Sequencing-grade trypsin was purchased from Promega (Madison, WI). Ultrapure water was prepared with a Milli-Q water purification system (Millipore, Billerica, MA). Other biochemical reagents and chemicals were of analytical grade.

### Cell line and cell culture

The NCI-H460 (human lung carcinoma), SMMC-7721 (human liver carcinoma), HCT-116 (human colorectal carcinoma), and U251 (human glioma) cell lines were obtained from the Cell Bank of Chinese Academy of Sciences Shanghai Institute of Cell Biology (Shanghai, China). NCI-H460 and SMMC-7721 cells were cultured in RPMI1640 supplemented with 10% FBS and 1% antibiotics mixture (streptomycin 100 μg/mL, penicillin 100 U/mL), HCT-116 and U251 cells were cultured in DMEM supplemented with 10% FBS and 1% antibiotics mixture (streptomycin 100 μg/mL, penicillin 100 U/mL). The cells were maintained at 37 °C with 5% CO_2_. The growth medium was changed every three days. Cells were passaged at 1:5 after they reached confluence.

### Cell growth inhibition assay

Cell viability was measured using the MTT assay (Xie et al. 2010). All cells were seeded in 96-well plates at a density of 4 × 10^3^ cells/well, incubated for 24 h at 37 °C with 5% CO_2_ and treated with A3, PSD, B4, R13, PSA, HC, HD, HG, and cisplatin at various concentrations (2.5–166 μM) in triplicate. After 48 h of incubation, 20 μL 5 mg/mL MTT was added and the cells were incubated for further 4 h at 37 °C with 5% CO_2_. Furthermore, formazan crystals were dissolved by adding 100 μL of DMSO. The optical density (OD) values were determined at 490 nm absorbance using a microplate reader (SpectraMax i3, Molecular Devices, CA) and presented as relative cell viability. The cell viability was calculated as follows: cell viability (%) = (A_490_ sample − A_490_ blank/A_490_ control − A_490_ blank) × 100%. The tests were repeated at least three times, independently.

### Evaluation of drug combinations

NCI-H460 cells were seeded in 96-well plates at a density of 4 × 10^3^ cells/well and were treated with PSD, R13, and PSA at various concentrations (2.5–166 μM) in triplicate. The activity level of these drugs alone against NCI-H460 cells was measured using the MTT assay as described above. The IC_50_ values obtained from single-drug cell viability assays were used to design the subsequent drug combination experiments.

Based on the IC_50_ values of PSD, R13, and PSA, CalcuSyn software (Biosoft, Cambridge, UK) (http://www.biosoft.com/w/calcusyn.htm) performed multiple drug dose-effect calculations using the Median Effect methods described by Chou–Talalay. The median-effect equation correlates the ‘Dose’ and the ‘Effect’ in the simplest possible form: fa/fu = (*D*/*D_m_*)*^m^*. *D* is the dose of the drug, *D_m_* is the median-effect dose signifying the potency, fa is the fraction affected by the dose, fu is the fraction unaffected, m is an exponent signifying the dose-effect curve. The *D_m_* and *r*-values for PSD, R13, and PSA were performed using CalcuSyn software.

As Chou–Talalay requires cytotoxic agents to be used at a fixed-dose ratio, cells were treated with the combination of PSD, R13, and PSA at *D_m_* as an intermediate concentration and used constant ratio 1:1.5 dilution of the highest concentration of drug combination to generate the dose range. Simultaneously, each drug in the combination was tested alone in the same manner. The dose-response experiments single drug alone and in combination was determined on NCI-H460 cells using the MTT assay as described above. A synergistic analysis was performed using CalcuSyn software again. The CalcuSyn software generated the combination index (CI) table for combination after the dose and IC_50_ values were entered. CI equation can be expressed as: CI=$\sum_{j=1}^{n}{\left(\mathrm{fa}\right)}_{j}/{\left(\mathrm{fu}\right)}_{j}.$ Fa is the fraction affected by the dose, fu is the unaffected fraction (therefore, fa = 1 − fu) (Chou 2006). Computer programmes based on the median effect plot parameters and CI equation were used to analyze synergistic, additive, or antagonistic effects in this study. CI < 1, CI = 1 and CI > 1 indicated synergism, additive effects, and antagonism, respectively. A confidence interval of 0.1–0.3, 0.3–0.7, 0.7–0.85, 0.85–0.90, and 0.90–1.10 indicated strong synergism, synergism, moderate synergism, slight synergism, and nearly additive, respectively (Chou 2010). Detailed procedures for using CalcuSyn software for automated dose-effect analysis for parameters of each drug and its combinations for quantitation/simulation of synergism or antagonism are given in the user guide for CalcuSyn software (Tallarida 2010; Zhang et al. 2016). Experiments were repeated in triplicate.

### Flow cytometric cell apoptosis analysis

NCI-H460 cells were seeded in 6-well plates (2 × 10^5^ cells/well) and incubated. After 24 h, the cells were treated with a combination of 6.0 µM PSD, 5.2 µM R13 and 9.0 µM PSA (at a concentration inducing 75% inhibition rate in combination) or equally effective individual drug for 24 h. The cells were washed twice with fresh PBS and trypsinized. Binding buffer containing 5 μL of Annexin V-FITC and 5 μL of PI was added to the cell suspension, followed by incubation for 15 min on ice in dark. The samples were analyzed by flow cytometry (Gallios, Backman, IN) as instructed by the manufacturer.

### DAPI staining for apoptosis

The morphological changes in cells were assessed using the DAPI Staining Kit. NCI-H460 cells were cultured in 12-well plates (2 × 10^5^ cells/well) and exposed to PSD, R13, and PSA alone and in combination for 24 h. The cells were then fixed with 4% paraformaldehyde for 20 min at 4 °C and incubated with DAPI for 20 min in dark after being washed with PBS twice. Images of the cells were captured using an inverted fluorescence microscope (Leica, Microsystems GmbH, Wetzlar, Germany). Bright-blue fluorescence and condensed nuclei indicated apoptotic cells.

### Proteomic analysis

#### Cell protein preparation and trypsin digestion

NCI-H460 cells were seeded in a 10 cm dish (2 × 10^6^ cells/dish) and treated after 48 h with a combination of 6.0 µM PSD, 5.2 µM R13, and 9.0 µM PSA (at a concentration inducing 75% inhibition rate in combination). The cells were washed two times with PBS and lysed using RIPA containing 1 mM PMSF. The plate was placed on ice for 5 min and was occasionally swirled for uniform spreading. The lysate was collected and transferred to a 1.5 mL microcentrifuge tube. The pellet was sonicated, and then the samples were centrifuged at 14,000 *g* for 15 min. The concentration of proteins was determined using the BCA protein assay kit according to the manufacturer’s instructions, and the cleared lysates were stored at −80 °C for further use.

Total protein (250 μg) was drawn from each sample. The proteins in each sample were reduced with DTT (a final concentration of 10 mM) at 37 °C for 1 h and were subsequently alkylated with IAA (a final concentration of 50 mM) at 25 °C for 1 h in dark. The sample solutions were then added with pre-chilled (−20 °C) acetone to ensure the ratio of the original volume to the added acetone was 1/5. Vortex tube and incubate at −20 °C overnight to precipitate proteins. Centrifuged at 14,000 *g* for 15 min, and then discarded the suspension. Subsequently, 1.5 mL pre-chilled (−20 °C) 100% acetone was added to wash the sediment and centrifuged at 14,000 *g* for 15 min thrice. Each sample was air-dried for 2–3 min and diluted with 500 μL NH_4_HCO_3_ (50 mM). The sample was then digested using a modified filter-aided sample preparation method with sequencing-grade trypsin at an enzyme to protein ratio of 1:50 and incubated at 37 °C for 12 h. The digestion of each extract was stopped by adding 10 μL of 0.1% TFA (Wiśniewski et al. 2009). The released peptides were then desalted using the SPE C18 cartridges (Thermo Fisher Scientific, Waltham, MA) and lyophilized under vacuum prior to nano HPLC-MS/MS analysis. We used three biological replicates to assess the accuracy, reproducibility, and reliability of the identification of proteins.

#### Nano HPLC-MS/MS analysis

Peptides were analyzed on a nanospray LTQ XL Orbitrap MS (Thermo Fisher Scientific) operated in a data-dependent acquisition mode with the installed XCalibur software version 2.0.7 (Thermo Fisher Scientific). The peptides were dissolved in 0.1% formic acid and centrifuged at 14,000 *g* for 10 min; the final peptide concentration in each fraction was 0.2 μg/μL. The peptides (10 μL) were loaded onto an Acclaim PepMap100 C18 75 μm × 2 cm trap column (Thermo Fisher Scientific) using an autosampler of the Ultimate 3000 NanoLC system (Thermo Fisher Scientific), and then eluted on an Acclaim PepMap C18 75 μm × 15 cm analytical column (Thermo Fisher Scientific).

Peptides were separated under a linear 4–90% acetonitrile gradient (in 0.1% formic acid). The elution process was controlled in 120 min at a flow rate of 250 nL/min. The peptides ionised by nano-electrospray at a voltage of 1.7 kV. Full-scan mass spectra were acquired using the LTQ Orbitrap mass spectrometer over 400–2000 *m*/*z* with a resolution of 60,000. A lock mass function was used for high mass accuracy. The five most intense ions per MS scan with a charge state of more than + 2 were sequentially isolated and selected for the MS/MS analysis in the order of their signal intensity (highest intensity first with a signal intensity threshold set to 5000) with a dynamic exclusion duration of 60 s. Precursor ions were activated using 35% normalized collision energy at the default activation q of 0.25. The *m*/*z* scan range was 300–1800 and 100–1800 Da for MS1 and MS2 scans, respectively. Three technical replicates per sample were performed.

#### Database searching and bioinformatic analysis of proteomics data

Peptide information analysis was performed using Proteome Discoverer Software (version: 1.4, Thermo Fisher Scientific). The identified proteins were analyzed using the SEQUEST search engine according to annotations from the UniProt database (Taxonomy: Homo sapiens, 20,342 sequences). For protein identification, a maximum allowed peptide mass error of 10 ppm, with allowance at most two missed cleavages in the trypsin digests. The acquired results were exported into the SIEVE software (version: 2.1.3, Thermo Fisher Scientific) for differential analysis. Proteins exhibiting differential expression were analyzed using DAVID Bioinformatics Resources 6.8 online (https://david.ncifcrf.gov/) (Huang et al. 2007). Statistical analysis was performed using R software environment. The biological functions and related pathways were analyzed based on the information available at the GO (Gene Ontology, http://www.geneontology.org/) and KEGG databases (Kyoto Encyclopaedia of Genes and Genome, https://www.genome.jp/kegg/), respectively. Functional protein-protein interaction (PPI) network analysis and candidate protein prediction were performed using interaction data from the STRING (Search Tool for the Retrieval of Interacting Genes/Proteins, http://string-db.org/) database.

### Western blotting

For western blotting analysis, cells were harvested and lysed in RIPA containing 1% PMSF for 30 min on ice. The lysate was collected and used to determine the concentration of proteins using the BCA protein assay kit. Proteins (30 μg) were separated by 10% sodium dodecyl sulphate polyacrylamide gel electrophoresis (SDS-PAGE) and transferred on to polyvinylidene fluoride (PVDF) membranes. After blocking with 5% non-fat dry milk dissolved in TBST (20 mM Tris-HCl, 150 mM NaCl, and 0.1% Tween-20, pH 8.0), the PVDF membrane was washed three times with TBST for 2 h at room temperature. The membranes were incubated with primary antibodies overnight at 4 °C, and then with the respective secondary antibodies for 2 h at room temperature. The target protein bands were detected using Bio-Ra Chemi Doc.XRS (Bio-Rad, Hercules, CA).

### Statistical analysis

Statistical analyses were performed using SPSS 19.0 software (SPSS Inc Chicago, IL). All data are expressed as mean ± SD. The statistical significance was evaluated using a one-way ANOVA and Duncan’s test (two-sided) was used to determine the statistical significance levels (*p* < 0.05 and *p* < 0.01) between control and drug-treated groups.

## Results

### Comparison of the inhibitory effects of various monomers against the four tumour cell lines

**Figure 2.**
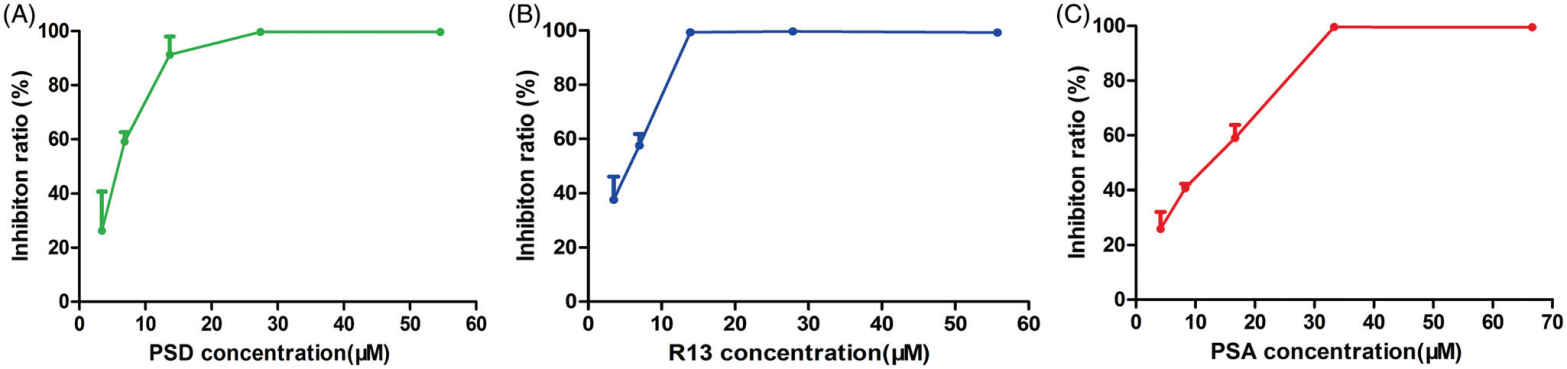
The inhibitory effects of PSD, R13, and PSA alone on NCI-H460 cells by MTT assay. (A) Inhibition rate of PSD on NCI-H460 cells. (B) Inhibition rate of R13 on NCI-H460 cells. (C) Inhibition rate of PSA on NCI-H460 cells. The results are representative of three experiments; the data are expressed as mean ± SD.

The results indicated that the anti-proliferative effect of PSD, R13, and PSA was higher than that of other monomers on the four tumour cell lines (Table 1). Particularly, PSD, R13, and PSA significantly suppressed the proliferation of NCI-H460 cells in a dose-dependent manner (Figure 2). As shown in Table 1, the IC_50_ value of PSD, R13, and PSA were 5.6, 5.1 and 10.5 µM for NCI-H460 cells, respectively.

**Table 1.** IC_50_ values of A3, PSD, B4, R13, PSA, HC, HD, HG, and cisplatin for four types of cancer cells (µM).

Cell line	IC_50_ values (Mean± SD)								
	A3	PSD	B4	R13	PSA	HC	HD	HG	Cisplatin
NCI-H460	107.1±5.4	5.6±0.3	56.8±5.2	5.1±0.1	10.5±0.6	34.1±6.7	39.3±6.7	36.6±0.8	10.3±0.3
SMMC-7721	221.5±12.5	8.7±0.9	165.4±52.8	2.9±0.1	10.1±0.9	53.1±13.0	60.1±16.1	143.8±49.7	8.0±0.3
HCT-116	53.5±4.2	3.6±0.0	43.0±0.8	3.9±0.1	9.9±0.5	35.0±2.1	40.2±4.2	97.1±8.0	13.6±0.3
U251	80.2±7.9	8.5±0.5	100.5±16.7	7.0±0.5	10.9±0.5	40.2±6.5	148.7±52.5	99.2±3.6	44.6±7.6

### Synergistic inhibition of NCI-H460 cell proliferation by the combination of PSD, R13, and PSA

**Figure 3.**
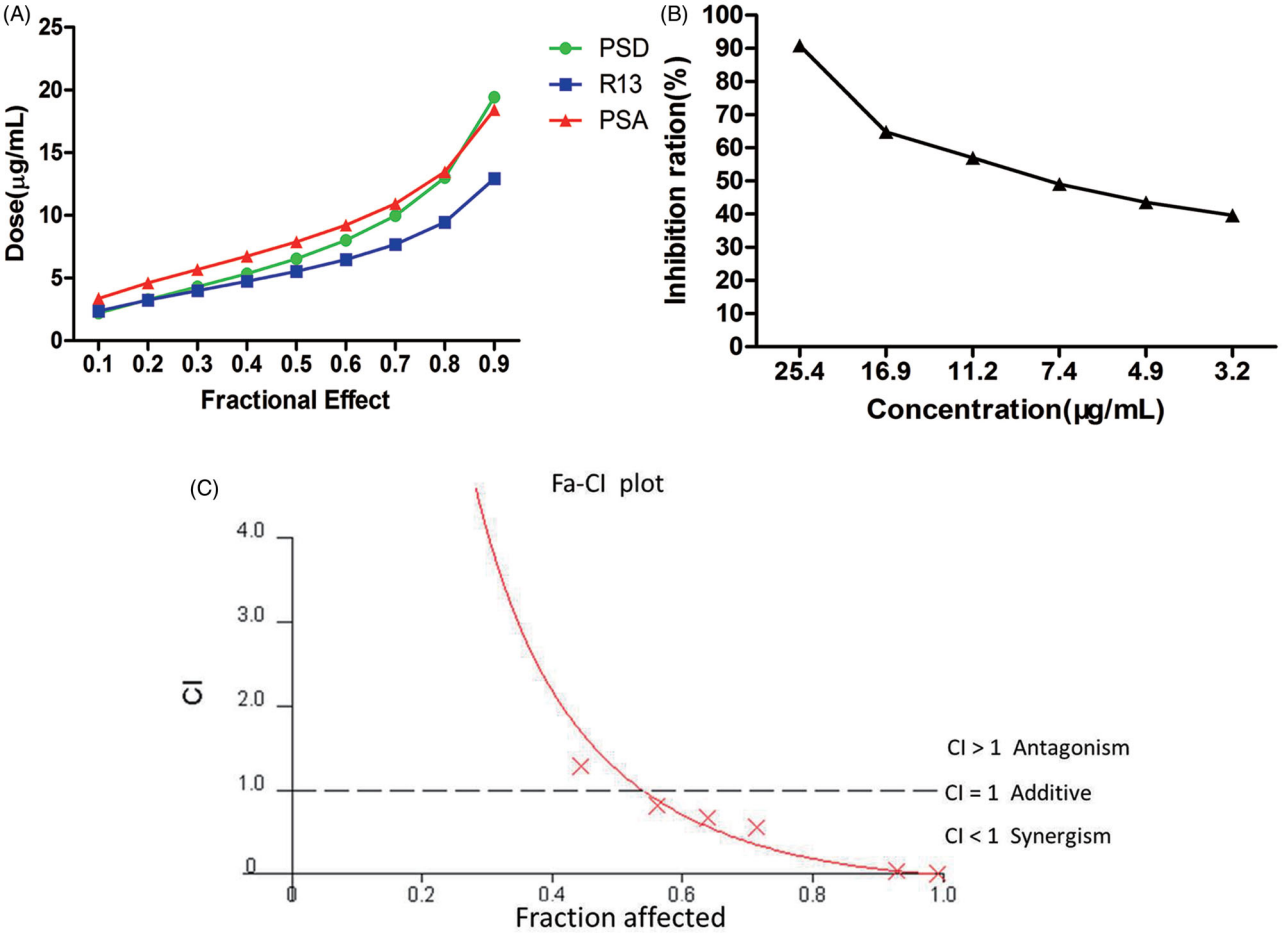
Synergistic anti-proliferation effect of PSD, R13, and PSA against NCI-H460 cells. (A) The Fraction affected (Fa)–Dose curve of PSD, R13, and PSA was analyzed using CalcuSyn 3.0. (B) The inhibitory effects of the combination of PSD, R13, and PSA against NCI-H460 cells using the MTT assay. (C) Fraction-affected (Fa)–Combination index (CI) analysis on NCI-H460 cells treated with the combination of PSD, R13, and PSA; CI < 1, CI = 1, and CI > 1 indicated synergism, additive effects, and antagonism, respectively. The results are representative of three experiments; the data are expressed as mean ± S.D.

To determine the median effect concentration of PSD, R13, and PSA, the cells in the logarithmic phase were treated simultaneously with varying concentrations of PSD, R13, and PSA and compared with the untreated control cells. The *D_m_* value of PSD, R13, and PSA was 6.54, 5.53, and 7.88 µg/mL for NCI-H460 cells, respectively, as determined by CalcuSyn analysis (Figure 3(A)). The drugs at these concentrations were then used for further analyses. To obtain the synergistic combination dosage, the cells were treated with a combination of drugs at a ratio of 6.54:5.53:7.88 (PSD, R13 and PSA) and evaluated using the MTT assay. It was obvious that the combination of PSD, R13 and PSA can significantly suppress the proliferation of NCI-H460 cells in a dose-dependent manner and the *D_m_* value of the combination was 3.72 µg/mL, which indicated that the three monomers from *P. chinensis* had multi-component synergistic antitumour characteristics (Figure 3(B) & Table 2). In addition, fraction affected (Fa) – combination index (CI) curve showed that the combination of PSD, R13, and PSA had a synergistic effect (CI < 1) at Fa > 0.55 by CalcuSyn analysis of proliferation inhibition data (Figure 3(C) & Table 3). The dosage determined using CalcuSyn analysis for the combination was 6.0 µM for PSD, 5.2 µM for R13, and 9.0 µM for PSA at CI ＝ 0.27. Therefore, 6.0 µM for PSD, 5.2 µM for R13 and 9.0 µM were determined as the combination concentration for further experiments.

**Table 2.** *D_m_* values of PSD, R13 and PSA alone and in-combination on NCI-H460 cells (µg/mL).

Cell line	*D_m_*			
	PSD	R13	PSA	PSD + R13 + PSA
NCI-H460	6.54	5.53	7.88	3.72

**Table 3.** Fraction-affected (Fa)–Combination index (CI) values for PSD, R13 and PSA in combination on NCI-H460 cells.

PSD (μM)	R13 (μM)	PSA (μM)	CI
3.79	2.40	5.67	1.63
4.38	3.77	6.55	0.93
5.09	4.38	7.62	0.52
6.06	5.21	9.06	0.27
7.61	6.55	11.39	0.10
11.76	10.12	17.59	0.02

### Induced apoptosis by the combination of PSD, R13, and PSA

**Figure 4.**
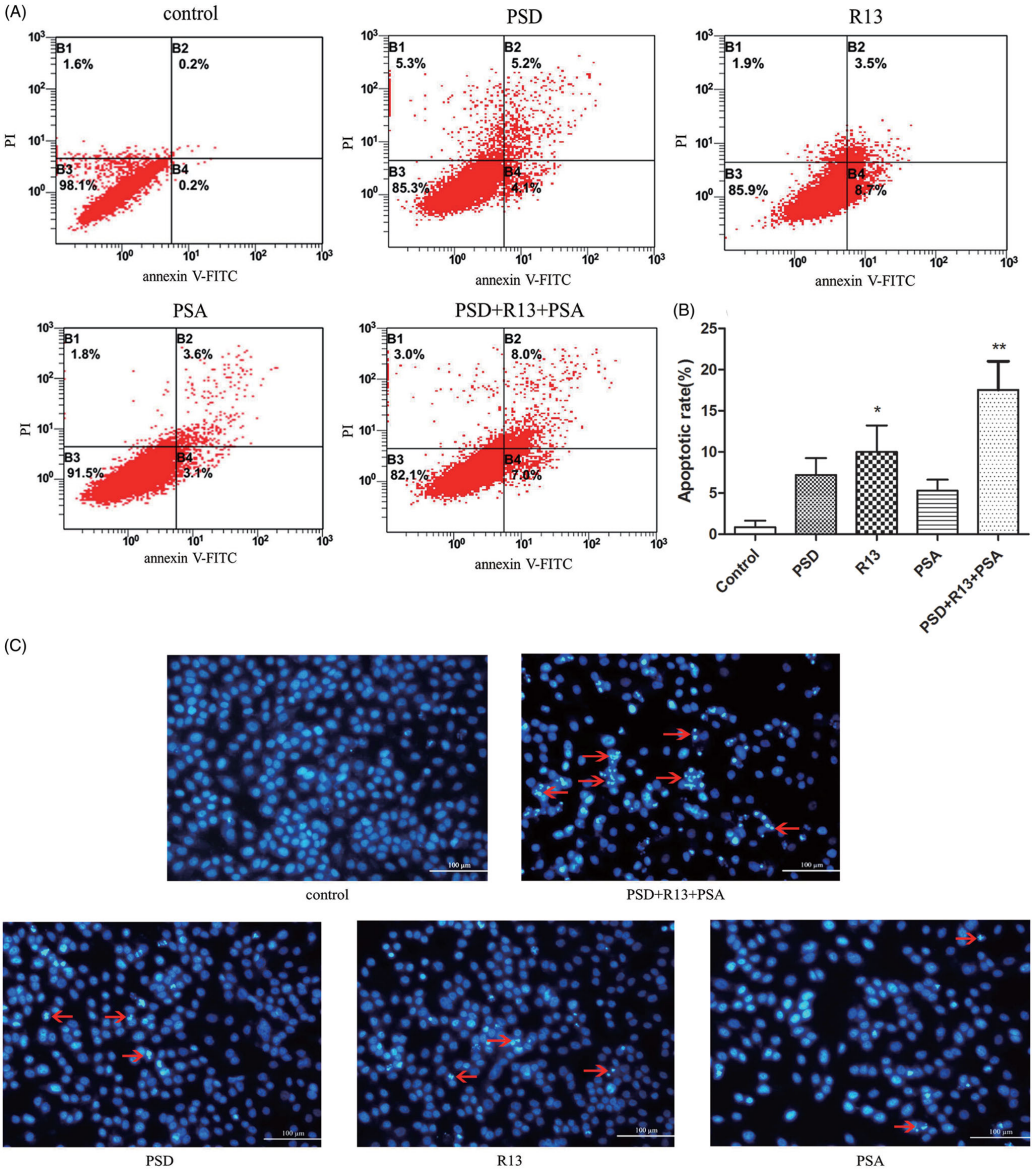
Apoptotic effects of PSD, R13, and PSA alone or in combination against NCI-H460 cells. (A) Flow cytometry histograms of cell apoptosis distribution after treatment with PSD, R13, and PSA alone or in combination. Early apoptotic cells were Annexin V+/PI − and late apoptotic cells were Annexin V+/PI+. (B) Quantitative analysis of apoptosis of cells is shown in (A). (C) DAPI staining of PSD, R13, and PSA alone or in combination in NCI-H460 cells. The presence of bright-blue fluorescent and highly condensed or fragmented nuclei represented apoptotic cells (×200). Arrows indicate apoptotic characteristics of treated cells. The results are representative of three experiments; the data are expressed as mean ± S.D. (*p < 0.05 vs. control group, ** *p* < 0.01 vs. control group).

The data showed that the apoptosis rate of each monomer group was higher than that of the control group, and that of the combination treatment group was considerably higher than that of the control group and monomer group (Figure 4(A,B)).

Additionally, the number of cells with the abnormal margin of the nucleus and concentrated chromatin stained bright blue were significantly increased after treated with the combination of PSD, R13, and PSA compared with those of untreated cells and cells treated with PSD, R13, and PSA individually (Figure 4(C)).

### Functional enrichment of the combination of drug-regulated proteins

To evaluate differentially expressed proteins, the fold-change threshold cut-off was set at >1.5-fold for increased accumulation and ˂0.67-fold for decreased accumulation.

**Figure 5.**
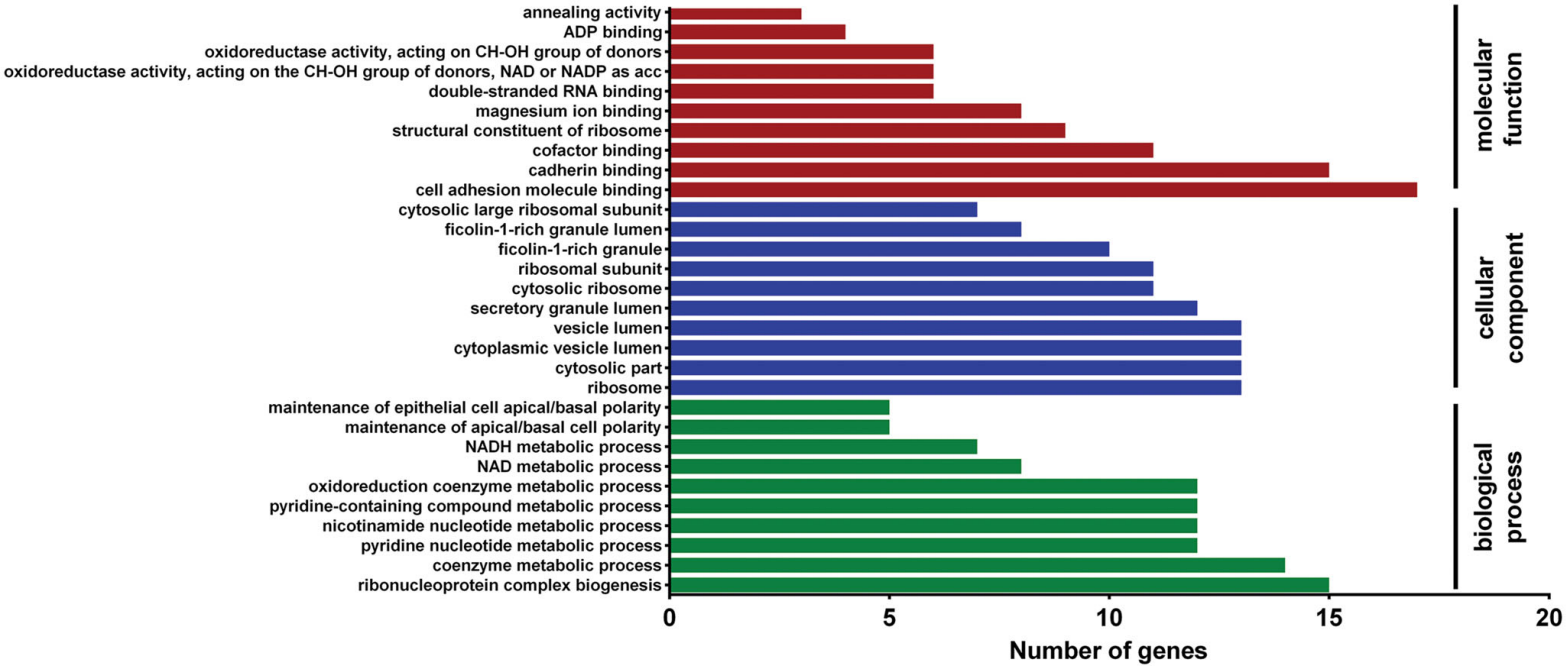
Gene Ontology (GO) enrichment analysis. An overview of top 10 significantly enriched terms in three categories: biological process (BP), cellular component (CC), and molecular function (MF). Number of proteins involved a process is shown in the *x*-axis. The cut-off of *p*-value was set to 0.05. Terms in the same category were ordered based on the *p*-values.

To obtain a biological view of the 116 differentially expressed proteins, enrichment of biological process (BP), cellular component (CC), and molecular function (MF) categories were analyzed using DAVID homology tool based on GO analysis. The BP analysis showed that the majority of identified proteins were classified into ribonucleoprotein complex biogenesis, coenzyme metabolic process, pyridine nucleotide metabolic process, and nicotinamide nucleotide metabolic process. Most of these proteins were located in the ribosome and cytosol part. The MF category revealed that most of these proteins are structural constituents involved in cell adhesion molecule binding and cadherin binding, and structural constituents of the ribosome (Figure 5).

### KEGG pathway analysis

**Figure 6.**
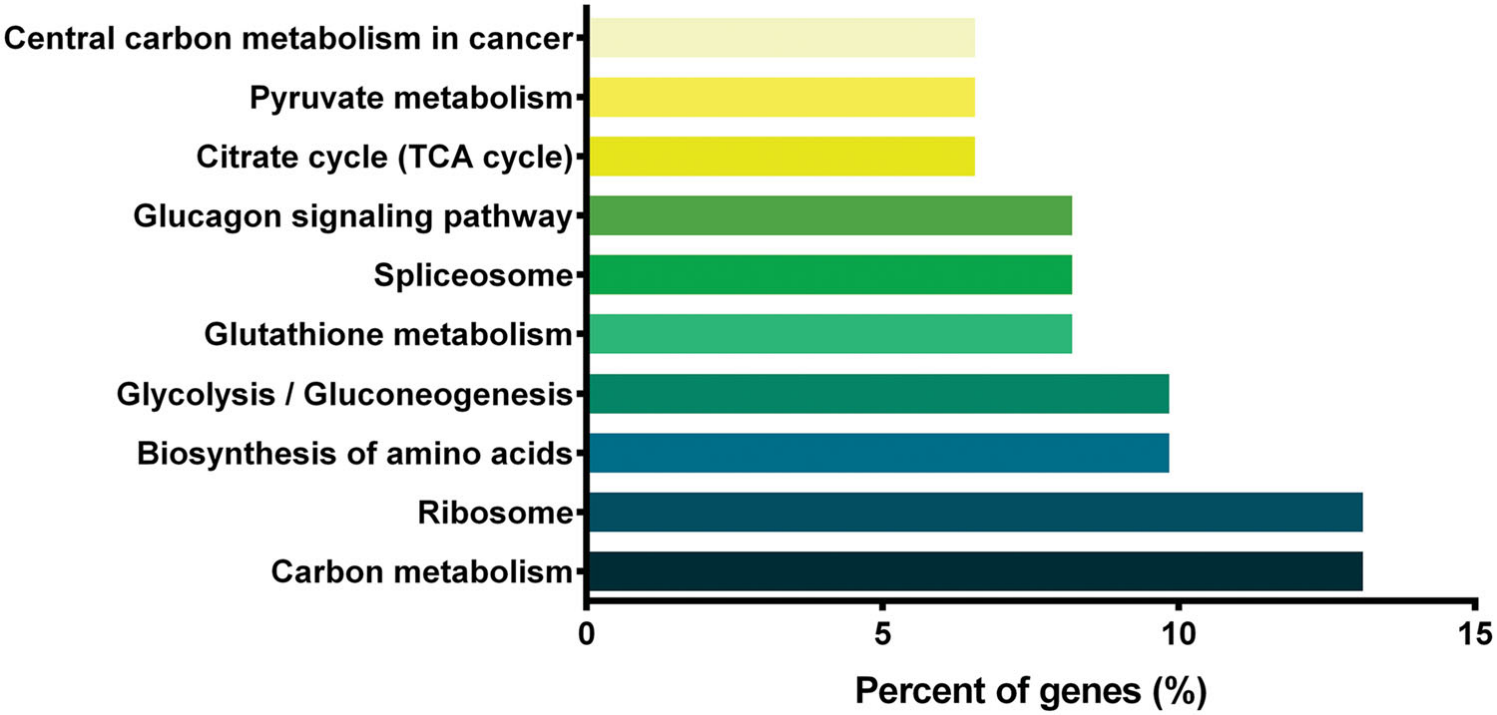
KEGG pathway enrichment analysis. Top ten pathways are ranked based on the *p*-value, and terms with *p* < 0.05 are statistically significant. The percent of genes is shown on the *x*-axis and the bars represent the number of involved genes.

The KEGG pathway analysis revealed 10 significant pathways with *p* ˂ 0.05. The top 10 pathways were Carbon metabolism (hsa01200), Ribosome (hsa03010), Biosynthesis of amino acids (hsa01230), Glycolysis/Gluconeogenesis (hsa00010), Glutathione metabolism (hsa00480), Citrate cycle (TCA cycle) (hsa00020), Pyruvate metabolism (hsa00620), Spliceosome (hsa03040), Central carbon metabolism in cancer (hsa05230), and Glucagon signalling pathway (hsa04988) (Figure 6).

### PPI network analysis

**Figure 7.**
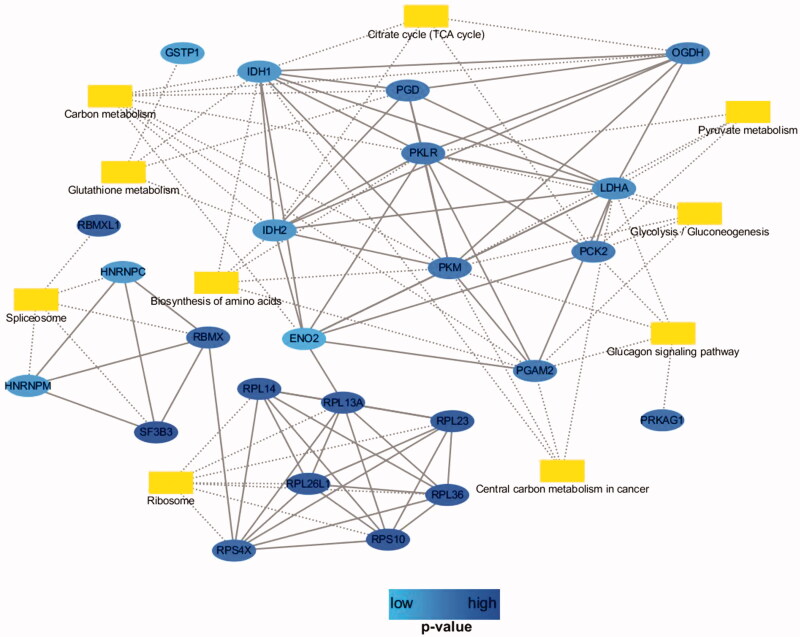
Protein–protein interaction network (PPI) analysis. The network was based on PPIs, and KEGG pathway and biological process enrichments. Blue circle nodes represent genes/proteins. Yellow rectangles represent KEGG pathways. The interactions between genes/proteins are shown as solid lines and the relationship between genes/proteins and protein and pathway is indicated by dotted lines.

The PPI based on the STRING web database and expression pattern was visualized using Cytoscape 3.6.0 software. There were 24 nodes and 58 edges in the PPI network for the identified differentially expressed genes. In the PPI network, PKM, PGAM2, IDH1, PCK2, and LDHA had higher degrees. A protein with a higher degree indicates that it is highly interconnected with other proteins in the PPI network (Figure 7).

### Validation of protein levels by western blotting

**Figure 8.**
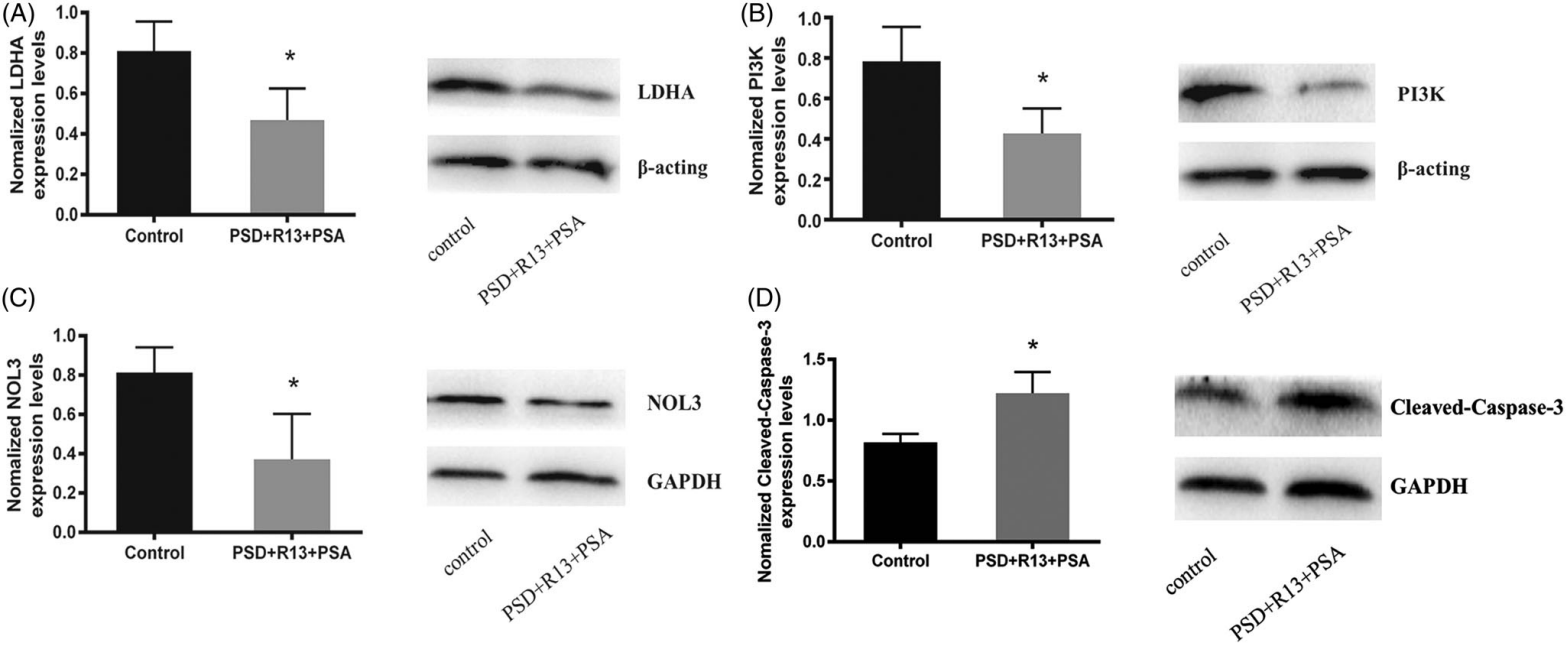
Validation of differentially expressed candidate proteins identified in the proteomics. Western blotting analysis shows the expression level of five proteins (LDHA, PI3K, NOL3, and caspase-3) in combination drug-treated and control cells (A-D). The data are expressed as mean ± S.D. of three independent experiments. *p < 0.05 compared with the control group.

The expression level of four dysregulated proteins was validated by western blotting in combination drug-treated and control cells based on the results of the MS analysis. Compared with those of the negative control, the expression of four proteins (LDHA, PI3K and NOL3) was significantly down-regulated (Figure 8(A–C)), whereas the expression of cleaved-caspase3 was significantly up-regulated in combination drug-treated cells (Figure 8(D)). This was consistent with the protein level obtained by MS analysis. It proved that the proteomic analysis outcome is reliable.

## Discussion

In the present study, we attempted to investigate the anti-proliferative and pro-apoptotic effects of *P. chinensis* against NSCLC cells at the proteomics level. First, we selected the high-efficiency antitumour active components extracted from the root of *P. chinensis* was extracted with alcohol and combined these active components. We found PSA, R13, and PSD significantly inhibited the proliferation of four tumour cell lines. To clarify the synergistic inhibition of NCI-H460 cell proliferation by the three components in combination, it is important to develop a scientific and reasonable evaluation method. At present, the theoretical basis for evaluating the interaction between drugs mainly includes mathematical methods, receptor theory, and biostatistics. Based on the mathematical model established by Chou–Talalay (Kuhlmann 1994; Chou 2006), CalcuSyn software can be used to calculate the *Dm*, *m*, and CI values, and develop inhibition rate-combination index (Fa–CI) curve by inputting the dose-effect values of single and combination drugs. Therefore, CalcuSyn software is now widely used to evaluate drug interactions, especially for evaluating the combined use of antitumour drugs (Tallarida 2010). Yim et al. (2006) found that a combination of two drugs can inhibit the growth of cervical cancer cells by affecting the death receptor pathway and mitochondrial pathway, using Chou–Talalay principle to combine 5-fluorouracil and cisplatin. In the present study, CalcuSyn software was first used to determine the compatibility ratio of the three monomers, followed by the combination of the three monomers for inhibiting the proliferation of tumour cells. Based on the dose-effect relationship data obtained using the MTT method, we could qualitatively and quantitatively describe the effects of the drug combination using CalcuSyn software. The results showed that the *D_m_* values of the drug combination were smaller than those of the single monomers, which indicated that the three monomers from *P. chinensis* had multi-component synergistic antitumour characteristics.

Second, the proteomics method was employed to analyze the potential signal pathways and molecular targets in NCI-H460 cells after combination treatment, and pathway and network analyses were performed. Proteomics is useful for revealing the mechanism of diseases at the proteomic level. It is also a powerful tool to reveal the potential targets or biomarkers by evaluating the targets of pharmaceutical products and analyzing the major components of physiological pathways (Ong and Mann 2006). In this study, the results showed that 116 proteins were differentially expressed in NCI-H460 cells after combination drug treatment. Bioinformatic analysis revealed that these proteins are involved in several BPs, and the majority of the identified proteins were classified into ribonucleoprotein complex biogenesis, coenzyme metabolic process, pyridine nucleotide metabolic process and nicotinamide nucleotide metabolic process, pyridine-containing compound metabolic process, oxidoreduction coenzyme metabolic process, and NAD metabolic process. These proteins were involved in multiple KEGG pathways and interacted with each other to form a PPI network. Carbon metabolism, ribosome, biosynthesis of amino acids, glycolysis/gluconeogenesis, glutathione metabolism, citrate cycle (TCA cycle), pyruvate metabolism, and spliceosome were the important enriched pathways.

Cancer cells are shown to undergo characteristic changes in their metabolic programmes to support growth and survival. Carbon metabolism is the most important metabolism in tumour cells. In the present study, some proteins PKM, IDH1, and LDHA related to carbon metabolic pathways and apoptosis initiation were significantly down-regulated after combination drug treatment. Studies have shown that PKM2 is the key enzyme of the aerobic glycolysis of the tumour cells as one of the four isoforms of PKM, which is known as a regulator of glycolysis in malignant tissues. PKM2 exists as a dimer in cancer cells, high expression of PKM2 leads to anabolic metabolism of glucose for macromolecular biosynthesis, which benefits cancer cell proliferation and tumour growth. High expression of PKM2 contributes to the aerobic glycolysis and promotes the growth of tumours. Therefore, PKM2 plays an important role in regulating tumour cell metabolism (Israelsen and Vander Heiden 2015; Dayton et al. 2016). Isocitrate dehydrogenase (IDH) is a metabolic enzyme converting isocitrate to α-ketoglutarate and consists of three isoforms. Studies have shown that the down-regulation of IDH1 altered carbon metabolism in the cytoplasm leading to mitochondrial respiratory depression, which ultimately affects tumour development and progression (Li and Zhao 2013). Lactate dehydrogenase A (LDHA) is an important catalytic enzyme in the regulation of glycolysis, which has been observed abnormally expression in many human cancers, such as pancreatic cancer and hepatocellular carcinoma (Sheng et al. 2012; Shi et al. 2014; Xian et al. 2015). Previous studies have demonstrated that reduction of LDHA induces oxidative stress and alters cellular energy metabolism, which ultimately contributes to cell death. LDHA knockdown or using an LDHA inhibitor induces an apoptosis ratio in cancer cells through induction of oxidative stress-mediated mitochondrial pathway apoptosis (Le et al. 2010; Wang et al. 2012; Zhai et al. 2013). In this study, the expression of LDHA was down-regulated after the combination of drug treatment. This showed that the combination of monomers from *P. chinensis* saponins might involve metabolism in NCI-H460 tumour cells.

In proliferating cells, ribosome biogenesis, and mitochondrial metabolism reprogramming are central processes for gene expression and macromolecular synthesis, which are inextricably associated with cell growth and division. In the present study, key nuclear proteins and other mitochondrial proteins were identified as downstream or upstream targets of the MAPK and Akt signalling pathways, such as EEF1D, HNRNPM, and EIF4H. A recent study showed that eukaryotic translation elongation factor 1 delta (EEF1D), another subunit of the eEF1 complex, mediates the elongation process after the translation initiation complex is formed. The expression of EEF1D was found to be up-regulated in human osteosarcoma tissues and cell lines. EEF1D knockdown inhibited osteosarcoma cell proliferation and plays a tumour promoting role by facilitating Akt-mTOR signalling pathways (Cheng et al. 2018). Heterogeneous nuclear ribonucleoproteins (HNRNPM) and Eukaryotic translation initiation factor 4H (EIF4H) plays an important role in regulating gene expression and translation initiation (Takino et al. 2015). Previous studies revealed that HNRNPM regulated genes were involved in PI3K/AKT/mTOR pathway, spliceosome and cellular junctions of Ewing sarcomas (ES) cells, suggesting that combined inhibition of the PI3K/AKT/mTOR pathway and HNRNPM activity may represent a novel approach for ES treatment (Passacantilli et al. 2017). In lung cancer cells, depletion of eIF4H enhances sensitization to chemotherapy, decreases cell migration and inhibits tumour growth. The result demonstrated that eIF4H plays an important role in the proliferation, migration, and invasion of cancer cells (Vaysse et al. 2015). It has been found that some compounds dysregulated translation and protein synthesis via the Ras/Raf/MEK/ERK (MAPK) or PTEN/PI3K/AKT pathways in cancer cells (Zhu et al. 2009; Pang et al. 2010). In the present study, PI3K was down-regulated in the combination of drug-treated NCI-H460 cells. Therefore, it was speculated that combination drug-induced apoptosis of NCI-H460 cells might be associated with the inhibition of RNA metabolism-related pathways, regulated through the Akt signalling pathway.

The apoptotic machinery is composed of two signalling pathways, comprising death receptors (extrinsic) and mitochondria (intrinsic) (Adams and Cory 2007). Intrinsic death stimuli directly or indirectly activate the mitochondrial pathway leading to the release of cytochrome c and the formation of the apoptosome complex consisting of cytochrome c, Apaf-1, and caspase-9 (Roderick and Cook 2008; Trachootham et al. 2009). Nucleolar protein 3 (NOL3) is an apoptosis repressor that blocks multiple modes of cell death. Unlike most apoptosis inhibitors that suppress either the extrinsic or intrinsic pathways, NOL3 has been reported to inhibit apoptosis through both extrinsic (death receptor) and intrinsic (mitochondrial/ER) pathways. NOL3 inhibits the extrinsic pathway through direct binding of its CARD to death domains in the cytoplasmic tail of the death receptor and in FADD, an adaptor molecule. These interactions preclude the assembly of DISC and abrogate extrinsic signalling. It also limits the amount of soluble caspase-8 available for DISC-mediated activation by interacting with caspase-8 in a mitochondria localization- and phosphorylation-dependent manner. NOL3 inhibits the intrinsic apoptotic pathway in response to a wide range of stresses, via its interaction with BAX resulting in BAX inactivation, preventing mitochondrial dysfunction and pro-apoptotic factor release (Gustafsson et al. 2004; Mercier et al. 2008; Stanley et al. 2017). The previous study demonstrates that transduced NOL3 protein protected against oxidative stress-induced cell death by inhibiting the intrinsic and extrinsic apoptotic pathways and suggests that transduced NOL3 protein inhibited H_2_O_2_-induced neuronal cell death through the regulation of apoptotic signal pathways (Eun et al. 2016). In agreement with the other studies, the result of this study shows that the low expression of NOL3 and high expression of cleaved-caspase3 in NCI-H460 cells after combination drug treatment. Although further study is merited in order to understand the precise mechanism, the results suggest that NOL3 might be a new target of combination treatment-induced apoptosis.

However, there are some limitations to the present study, and more research is needed to further confirm our findings. First, the expression of differentially expressed proteins identified in the current study needs to be validated by more experiments. Second, the differentially expressed proteins interactions within the PPI network, as well as the regulatory relationships between P. chinensis saponins and differentially expressed proteins, need to clarify. Furthermore, the antitumour effects of this combination should be investigated in more tumour cell lines and animal models in future studies.

## Conclusions

We demonstrated that the three monomers PSA, R13, and PSD of *P. chinensis* saponins can considerably inhibit the proliferation of four tumour cell lines. Specifically, we investigated the usefulness of the combination of PSA, R13 and PSD in potentiating antitumour effect and inducing apoptosis in NCI-H460 cells. The synergistic anti-proliferation mechanism of PSA, R13 and PSD was explored using a label-free proteomic approach. The results showed that the antitumour effect of the combination against lung cancer cells might be associated with the inhibition of carbon metabolism, biogenesis of ribosomes, and synthesis of proteins. Overall, these findings help investigate whether the multi-component of *P. chinensis* saponins have a synergistically antitumor effect, explore its underlying molecular mechanisms and provide a new strategy for cancer therapy by using *P. chinensis* saponins.
